# Fire-Shaped Nozzles to Produce a Stress Peak for Deformability Studies

**DOI:** 10.3390/polym14142784

**Published:** 2022-07-07

**Authors:** Alejandro Rubio, Marta López, Emilio J. Vega, María G. Cabezas

**Affiliations:** Departamento de Ingeniería Mecánica, Energética, y de los Materiales and Instituto de Computación Científica Avanzada (ICCAEx), Universidad de Extremadura, Avda. Elvas s/n, E-06006 Badajoz, Spain; arubiorg@unex.es (A.R.); mlopezfop@alumnos.unex.es (M.L.); mguadama@unex.es (M.G.C.)

**Keywords:** microfluidics, particle deformability, strain rate peak, fire-shaped nozzles, polymeric particles

## Abstract

Fire-shaped nozzles can be used to study the deformability of microcapsules, particles, or cells traveling in a flow. Though their geometry depends on the dimensions of the original glass capillary and the heating conditions, they all produce a strain rate peak approximately at the section where the diameter is 1.5 times the minimum. The intensity of this peak and the time from its position to the neck can be easily estimated from the flow rate and three geometrical parameters, without the need for any simulation. In the convergent region of these nozzles, it is possible to observe the evolution of the deformation. It is necessary to use a sufficiently long nozzle to produce the maximum deformation before the neck.

## 1. Introduction

Capsules consist of an outer membrane or shell that surrounds an inner medium. Their purpose is to contain, protect, transport, and/or deliver an active material. Artificial microcapsules are common in the medical, pharmacy, cosmetic, and food industries. Their composition and structure are usually customized for a particular application. Their flow behavior can be tuned by adjusting the composition and thickness of the shell [1]. Their break-up, or its prevention, is also relevant for some processes [2]. For drug delivery, the agent release at a target can be controlled by using multilayer shells with components that react to physical, chemical, or biological stimuli [3]. Red Blood Cells (RBCs) can be considered natural microcapsules. They are responsible for oxygen transport and its release to the body tissues. The outstanding deformability of their membrane allows them to flow through capillaries smaller than their diameter. RBCs’ physical properties are known to play a significant role in blood fluidity both in the bulk flow and microcapillaries [4]. The interest in their study has motivated the development of synthetic microcapsules and microparticles to mimic their behavior [5].

The mechanical properties of micrometric cells, capsules, and particles are crucial for performing their function. For that reason, several methods have been used for their measurement, and are still being developed, which provide complementary information (see Ref. [6] and references therein). Overall properties can be determined from particle suspensions by compression in a rheometer [5] or filterability studies [7], while single-cell measurements can be obtained by micropipette aspiration, atomic force microscopy, or optical tweezers. Additionally, microaspiration, which combines optical observation with ion current signal analysis, was recently proposed to improve the throughput of conventional micropipette aspiration [8]. Advances in microelectromechanical systems (MEMS) have allowed the creation of planar microfluidic devices for the fabrication, characterization, and even sorting and separation of these micro-entities. A single device can be designed to combine several functions, such as polymer microcapsules production and deformability and relaxation measurements [9] or cell elasticity measurement and separation [10].

Microfluidic devices have the potential to become common diagnosis tools for cell deformability (see [11,12] and references therein). The combination of these systems with high-speed imaging has enabled the study of the cell behavior in the flow [13,14]. In realtime deformability cytometry (RT-DC) [15] over 100 cells/s are analyzed. Constrictions, channels with sections smaller than the cell, allow the evaluation of the stiffness from its confined deformation or from the entry or transient times [16]. A decreasing size channel network was used to model the flow of RBCs through capillaries [17], and measure their deformability. The viscoelastic properties of breast cells have been calculated from their deformation under the compression caused by the walls in a confining microchannel [18]. The focus can also be set on the obstruction of the channel, as in the study of the effect of RBCs increasing rigidity at different stages of malaria infection [19]. A new micro-device based on microvascular occlusion were recently proposed to RBCs assessment [20,21], showing its clinical associations in sickle cell disease (SCD) [22]. The loss of RBC deformability during cold storage has been analyzed by using microfluidic capillary networks [23,24], showing more sensitivity than ektacytometry. In devices with sections larger than the cell, the particle deformation is caused contactless by flow stresses which depend on the channel geometry. In straight channels, the velocity gradient is perpendicular to the flow and the particle deforms due to the shear flow. In this case, the measurements are taken far enough from the entrance of the channel [15] so that the particle is not responding to the contraction effect. To reduce the flow resistance, RBCs’ shapes change to parachute or slipper-like depending on the flow conditions [13]. Cross-slots microchannels formed by two perpendicular channels with opposite inlet and outlet sections produce a flow with a stagnation point at the center. So, the particle is stretched by the extensional flow associated with the velocity gradient along the flow direction. Alternative cross-Section geometries [25] or the use of a biocompatible viscoelastic medium for the suspension [26] have been proposed to improve the performance of these devices. Channels with contractions also produce an extensional flow due to the velocity gradient along the flow direction. Hyperbolic convergent square-shaped channels are usually chosen to obtain a homogeneous extensional flow. They have been used for observing the evolution of the RBCs deformation along the channel for different flow rates [27], or the RBCs deformability reduction after heating [28], and to state that the cell deformation caused by extensional stress being much larger than the corresponding for a similar magnitude shear stresses [14,29].

The use of glass nozzles and capillaries is common for the construction of three-dimensional microfluidic assemblies, such as those for producing monodisperse droplets, particles or capsules [30,31]. The nozzles are typically fabricated by pulling or fire-shaping, producing the latter significantly shorter nozzles for the same diameter reduction. Fireshaped nozzles have convergent-divergent shapes. Their geometry depends on the heating conditions, and the dimensions of the original capillary used to produce it [32,33,34]. The contraction produces an extensional flow, and we recently proposed its use for studying the deformation of RBCs [35]. Our micro-device consists of a simple borosilicate micronozzle whose production is simple, fast, and low-cost by the flame polishing method when compared with other similar microfluidic devices previously mentioned, whose a time-consuming manufacturing process usually involves a soft lithography technique that requires a clean room environment. Moreover, our device allows the deformability assessment of hundreds of particles/cells in a continuous flow, in each experiment, instead of a single particle/cell.

In this work, we analyzed the flow through fire-shaped nozzles to understand the particle deformation measured with this device. We numerically studied the flow through nozzles of approximately the same neck diameter and significantly different shapes. All these nozzles produced a strain rate peak before the neck. So, they can be used to test the response of microparticles to this kind of stress. Our results show that regardless of their shape, the peak is applied at the section of diameter 1.5 times the neck one. Its intensity and time from its position to the neck can be estimated from the flow rate and a few geometrical parameters. We used flexible polydimethylsiloxane (PDMS) particles to measure the evolution of their deformation and the delay with respect to the stress applied. For deformability studies, nozzles should be sufficiently long to produce the maximum deformation before it crosses the neck.

## 2. Materials and Methods

### 2.1. Deformation and Size Measurements

**Figure 1:**
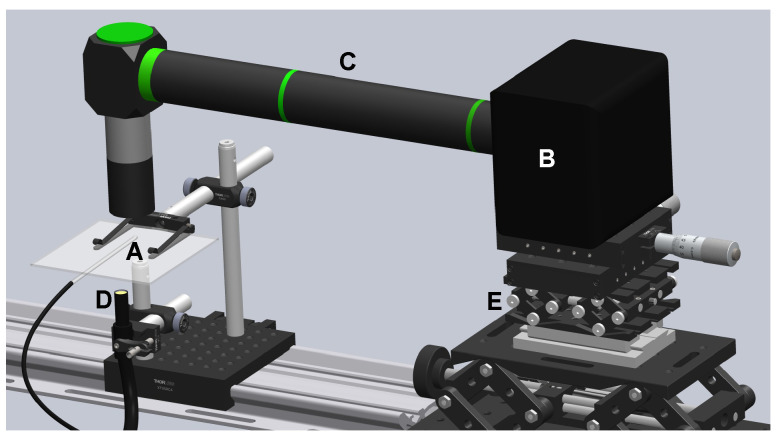
Setup for observing the particle deformation: (A) glass nozzle on the reference surface, (B) CMOS camera, (C) lenses, (D) light source, and (E) translation stage.

**Figure 2:**
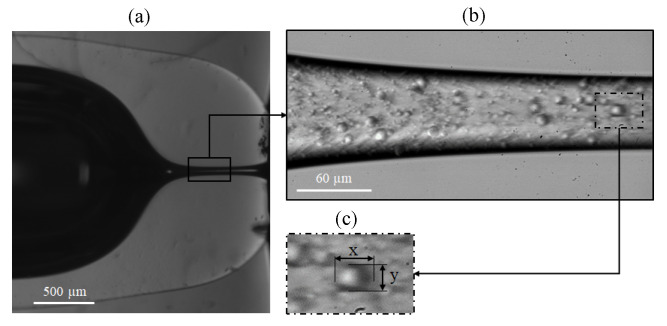
Experimental images used for the characterization of the nozzle shape (**a**), and to evaluate the particles deformation (**b**). Panel (**c**) shows the measurement region for the neck section, and the dimensions of the particle used in the deformation index.

Figure 1 shows the experimental setup used to observe the deformation of the flexible particles while flowing along the nozzle. The glass capillary with the nozzle (Figure 2a) was fastened to the sample holder surface (A). The nozzle end was submerged in a glycerol bath to minimize optical effects in the observation through the glass, while the opposite end was connected to a standard syringe pump using adequate polymer connectors and tubing (from Postnova, IDEX). We used a high-speed CMOS camera (Photron, Fastcam Mini UX50) (B), equipped with the corresponding lenses (C), and an optical fiber light source (D) to observe a 355 × 130 μm region around the nozzle neck (Figure 2b). The camera could be displaced horizontally and vertically using a triaxial translation stage (E) to focus the micro-entity. Images were acquired at 5000 frames per second, with an 11.11 μs exposure time and a magnification of 0.28 μm/pixel. To evaluate the deformation of the particles, we used the Deformation Index DI=(X−Y)/(X+Y), where *X* is the length of the particle along the nozzle/capillary axis and *Y* is the corresponding in the normal direction. In each experiment, 100 particles moving along the nozzle centerline (±5μm) were measured when crossing a particular section (±33μm). Figure 2c shows the measurement region at the neck section. The particle dimensions were obtained manually at the pixel level, and Chauvenet’s criterion was used to identify and reject outliers. The experimental procedure for one DI measurement from a sample of one hundred particles, from sample preparation to data analysis, took typically less than 2 h.

### 2.2. Fabrication and Characterization of the Nozzles

Glass nozzles were fabricated from commercial capillaries by fire-shaping. The tip of a rotating vertical capillary was introduced at the bottom of a lateral flame, which was produced by a Bunsen burner placed with its tube horizontal. The resulting nozzle geometry depends on the corresponding of the original capillary and on the heating conditions (position and time). When the capillary is heated at an outer position, the diameter reduction takes longer and spreads over a shorter length, therefore resulting in a shorter nozzle. Details on the setup and process can be found in [34].

**Figure 3:**
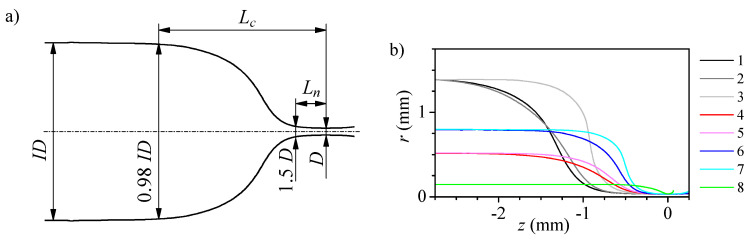
Characteristic geometrical parameters of the nozzle inner shape (**a**), and mean profile of the nozzles (**b**). In the latter, the origin z=0 is located at the nozzle neck, and the line colour indicates the nozzle number in Table 1.

We fabricated eight nozzles with the same neck diameter (D≈65μm) and significantly different shapes using different capillaries (inner diameter ID and outer diameter OD), and/or different heating conditions (radial $r_{h}$ and axial $z_{h}$ distance to the burner tube exit center, and heating time $t_{h}$) (see Table 1). To characterize their geometry, we took images of three different views of each nozzle (Figure 2a). By an image analysis procedure [33] we obtained the nozzle mean profile and calculated some geometrical parameters as the neck diameter *D*, the convergent length $L_{c}$ and the neck length $L_{n}$ (Figure 3a). The convergent length measures the region where the whole diameter reduction occurs, from 0.98×ID to *D*, while the neck length accounts for the final reduction, from 1.5×D to *D*.

The difference in the shape of the nozzles is readily appreciated in Figure 3b. When the original capillary is larger (higher ID), so is the desired diameter reduction, and due to the manufacturing process, the nozzle is longer and has a longer neck. The use of thin-wall capillaries significantly reduces the amount of glass involved in the shaping process and consequently the heating time; for example, nozzle 2 only needs about 25% of the corresponding for nozzle 1. However, it does not significantly affect the neck length. Choosing an outer heating position (higher $r_{h}$) in the flame allows for reducing the neck and nozzle lengths, especially for thin wall capillaries (see, for instance, nozzles 2 and 3). However, the fabrication time increases sharply which, in practice, establishes a limit to the heating position. Using smaller capillaries (lower ID) reduces the amount of glass involved in the shaping and the diameter reduction, which accelerates the shaping process. To keep the process under control, it becomes necessary to work outer in the flame. The effect of varying the inner capillary diameter and its wall thickness may compensate, and larger capillaries with thinner walls may result in shorter nozzles (see nozzles 4 and 6). Finally, the use of very thin capillaries (nozzle 8) produces very short nozzles, but the shaping process is very fast and has poor reproducibility [34]. In general, we conducted around seven experiments for each nozzle device.

### 2.3. PDMS Particle Suspension Preparation

The two-syringe membrane emulsification technique (2SME) [36] was used for fabricating the flexible PDMS particles. First, we prepared the mixture between the siloxane base (Part A) and the curing agent (Part B) (Dow Corning SYLGARD 184 Silicone Elastomer) with a ratio of 30:1 (wt% siloxane base to curing agent) and stirred it manually for ten minutes. Then, we loaded 1 mL of the PDMS precursor mixture in one syringe and 5 mL of distilled water with a surfactant (3 wt% sodium dodecyl sulfate, SDS) on the opposite. The addition of the surfactant prevents sedimentation and flocculation of the particles. The emulsion was produced with five back and forth flow cycles through a 10 μm pore size filter. To cure the PDMS, we placed the emulsion in a standard magnetic stirrer at 70 ${}^{\circ }$C for 3 h, and then we waited for 24 h to reach room temperature. The final particle proportion in the suspension was 1.65±0.24 wt% (calculated by drying different samples). For each experiment, we prepared the particles solution and then modified the liquid phase by adding Dextran 40 (10 wt%). The final suspension density and viscosity are 1048 kg/m${}^{3}$ and 0.0046 Pa·s. Dextran in the solution decreases sedimentation phenomena in the glass capillary, whereas the dye enhances visualization of the contours of the PDMS microparticles. On the other hand, the surfactant SDS avoids the formation of particle aggregates.

## 3. Results

### 3.1. Numerical Study of the Flow

We conducted numerical simulations using Ansys Fluent to analyze the strain rate resulting from the flow through the nozzles. We used the mean profile obtained from the images (Figure 3) to define their geometry and studied the flow of a liquid without particles with the properties shown in Section 2.3. The flow was regarded as axisymmetric. We imposed the non-slip condition on the solid walls and prescribed the inlet mass flow rate and the outlet pressure. We verified that the results were insensitive to the grid size.

**Figure 4:**
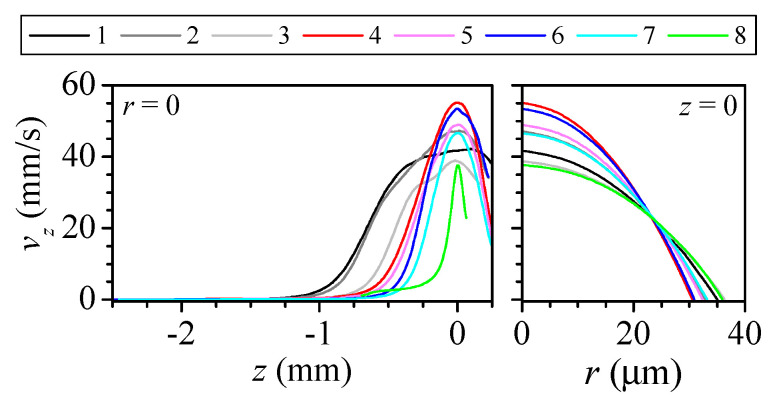
Axial velocity at the axis along the nozzle (**left**), and at the nozzle neck (**right**) for all the nozzles. The line colour indicates the nozzle number in Table 1.

The flow accelerates along the convergent region, and the velocity reaches a maximum at the neck, to decrease again downstream in the divergent region (Figure 4 left). As the Reynolds number Re = 4ρQ/(πμD) is below 0.5, the velocity profile at the neck section is almost parabolic (Figure 4 right). The difference between the maximum velocity at the neck and the corresponding for the parabolic profile is below 4% for nozzles 1–7, and rises to approximately 8% for nozzle 8.

**Figure 5:**
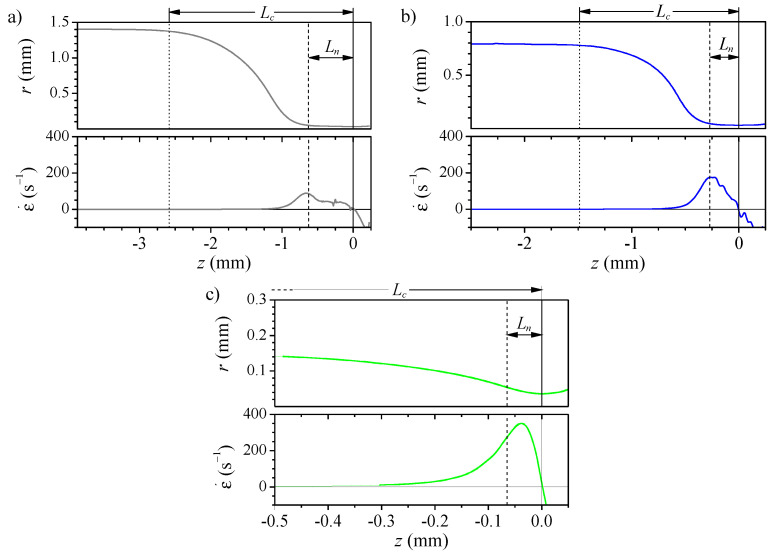
Shape and strain rate along the nozzle axis for nozzles 2 (**a**), 6 (**b**) and 8 (**c**) and Q=5μL/min. The vertical lines indicate sections of diameter *D*, i.e., the neck, (solid), 1.5 ×D (dashed), 0.98 ×ID (dotted). The latter is out of the field in (**c**).

**Figure 6:**
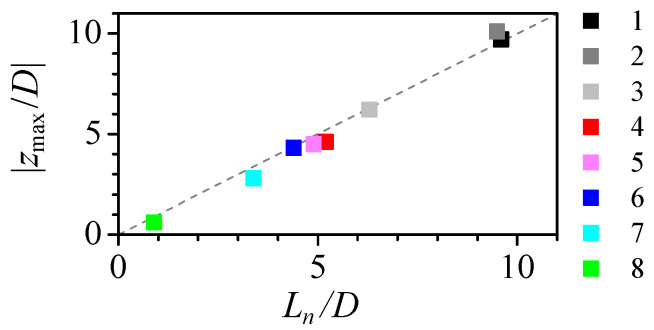
Maximum strain rate position $z_{\max}$ versus the neck length $L_{n}$ for Q=5μL/min. The colour indicates the nozzle number in Table 1. The dashed line has slope 1.

Figure 5 shows the shape and the strain rate $\dot{\epsilon }=\partial v_{z}/\partial z$ for three of the nozzles. The diameter reduction from the original capillary diameter ID to that of the neck *D* spreads over a long region. We use two distances to characterize the shape: the convergent length $L_{c}$, measured from the section of 0.98×ID diameter to the neck; and the neck length $L_{n}$ which measures the region where the final diameter reduction, from 1.5×D to *D*. For all the nozzles, the strain rate remains negligible for almost two-thirds of the nozzle length. Then, it shows a peak approximately at the section used to define the neck length. Finally, it falls to zero at the neck and negative on the divergent side. Nozzles produced from larger capillaries (Figure 5a), despite their larger diameter reductions, are longer and have a longer neck which results in a lower strain rate peak located farther from the neck. Figure 6 shows that the distance from the maximum strain rate to the neck is approximately the neck length for all the nozzles. Therefore, in fire-shaped nozzles and regardless of their shape, the peak position can be estimated to be at the section of 1.5 ×D diameter (used to evaluate $L_{n}$).

**Figure 7:**
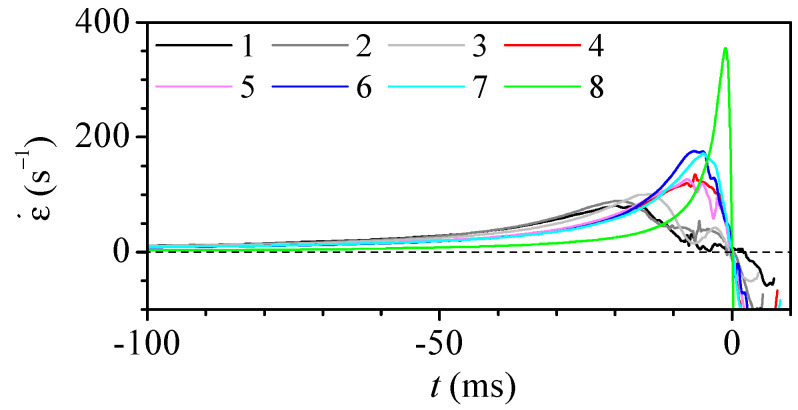
Strain rate along the nozzle axis versus the time to cross the neck for Q=5μL/min. The colour indicates the nozzle number in Table 1.

In deformability studies, the dynamical response of the particle to the stress caused by the fluid is measured. The deformation depends on the magnitude and duration of the stress applied. Figure 7 shows the temporal evolution of the strain rate suffered by a fluid particle along the axis. The time origin t=0 is set at the instant when the particle crosses the neck, so the (negative) time indicates the remaining time to reach the neck. As the velocity is higher at the neck, the stress peak is very brief (ranging from 20 to 0.1 ms) when compared to the time to travel the whole convergent region (from 0.2 to 25 s). The strain rate peak is more intense for the shorter nozzles, even when the diameter reduction is smaller.

**Figure 8:**
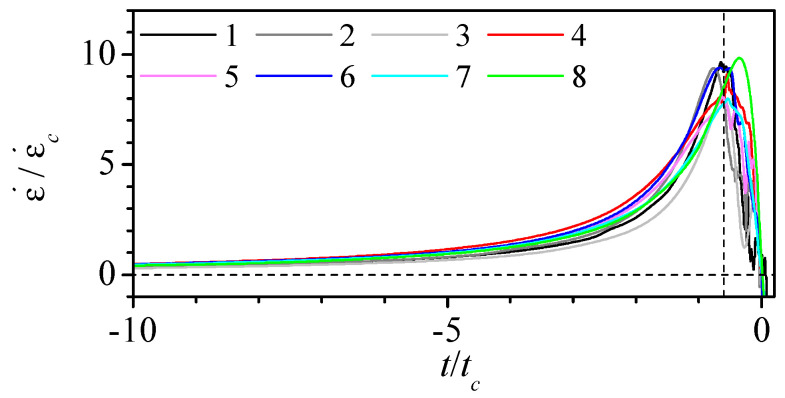
Dimensionless strain rate along the nozzle axis versus the dimensionless time to reach the neck for Q=5μL/min. The colour indicates the nozzle number in Table 1.

We used the mean strain rate in the convergent region $\dot{\epsilon_{c}}=v_{n}/L_{c}$ and the characteristic time $t_{c}=L_{n}/v_{n}$, where $v_{n}=4Q/(\pi D^{2}$) is the mean velocity at the neck, to calculate the dimensionless strain rate and time in Figure 8. Despite the significant difference in the geometry of the nozzles, the overlapping of the curves is remarkable. This is possible due to the nature of the shaping process, in which the heated glass surface tension force competes against the viscous force. The maximum strain rate ranges from 8 to 10 times the mean value $\dot{\epsilon_{c}}$ and it occurs approximately 0.6 times $t_{c}$ before the neck. Therefore, regardless of the nozzle shape, it is possible to estimate the maximum strain rate value and when it occurs just from the main geometrical parameters of the nozzle.

**Figure 9:**
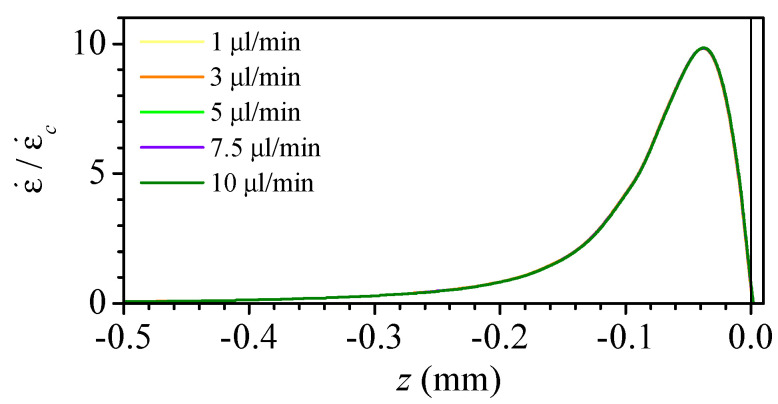
Dimensionless strain rate along the axis for nozzle 8 and different flow rates. The lines for all the flow rates overlap.

**Figure 10:**
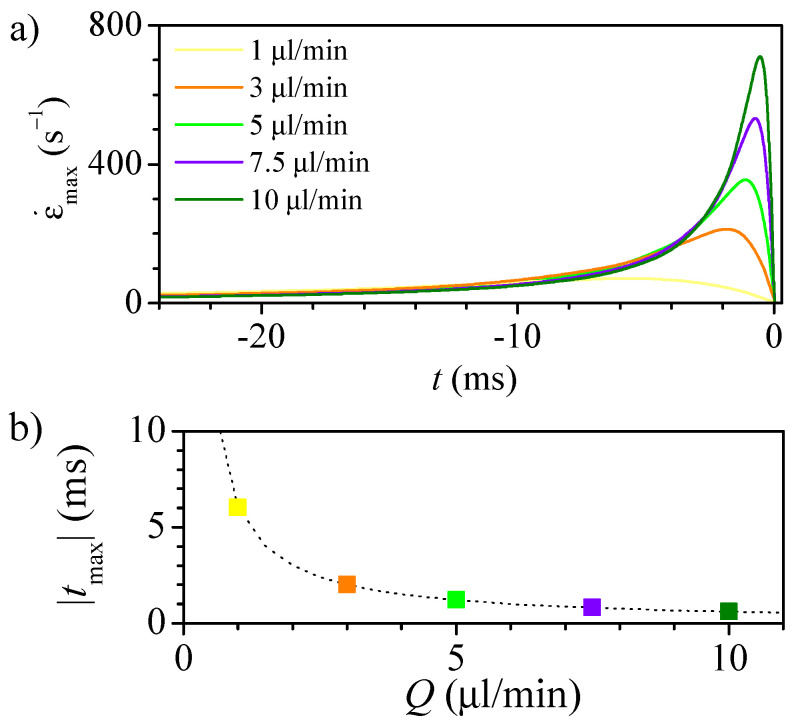
Strain rate versus time to reach the neck (**a**), and time from the maximum to the neck (**b**) for nozzle 8 at different flow rates. The dashed line in (**b**) correspond to the curve $6.05\times Q^{-1}$.

For a given geometry, the strain rate is proportional to the flow rate, and the position of the maximum remains at a fixed position (Figure 9). This linear relation confirms that the flow is practically developed at each nozzle section, which would allow using Poiseuille flow to estimate the velocity and strain rate at the nozzle axis. Figure 10a shows how the strain rate peak increases linearly with the flow rate, but it also becomes briefer and happens closer to the instant when the particle crosses the neck. As expected, the time from the maximum stress section to the neck is inversely proportional to the flow rate (Figure 10b).

### 3.2. Experimental Measurement of Particle Deformation

We used a suspension of PDMS particles to study their deformation while traveling in the flow through the nozzles. The particles were fabricated as described in Section 2.3. As the particles move along the nozzle, they are subjected to the variable stress exerted by the surrounding fluid, which causes their deformation. In this kind of flow, the stress source is mainly due to the axial acceleration of the fluid (the strain rate). Though the presence of the particles affects the flow, their volumetric ratio in the fluid is low and their size is small compared to the nozzle diameter. For that reason, the results of the numerical simulations may be adequate to estimate the stress on the particle, and therefore, they will be used to analyze the deformation measurements.

**Figure 11:**
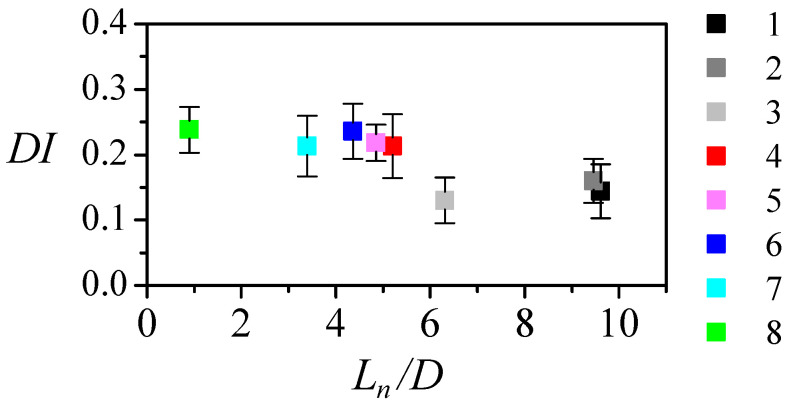
Particle deformation index DI measured at the neck versus the neck aspect ratio AR for the different nozzles. The symbol color indicates the nozzle number in Table 1. The flow rate was Q=5μL/min.

PDMS particles are known to show viscoelastic behavior. So, there is a delay in the particle response to the stress applied. Additionally, the deformation will depend on the stress, its duration, and the measurement position. It is easy to determine the neck section in the image, so the deformation index (DI) is usually measured when the particle crosses the neck. Note that, due to the converging-diverging shape, the strain rate is zero at the neck. So, the measured deformation is the response to the stress on the particle upstream. Figure 11 shows the deformation index at the neck versus the neck aspect ratio $AR=L_{n}/D$ for the different nozzles. The deformation index is about 0.15 for nozzles with AR>4.5 and rises to approximately 0.23 for AR<4.5. The numerical results showed that the lengths of the convergent region and of the neck are related to the strain rate peak, width and position. The lower deformation index measured for larger AR results from the application of a lower stress peak farther from the neck. The particle deforms less, and may even be recovering its shape when crossing the neck. The nearly constant deformation measured for AR<4.5 may be caused by different reasons. On the one hand, the AR reduction causes two opposite effects: it increases the stress but reduces its duration and the temporal distance to the neck. The particle deformation may not follow the stress peak because the stress is not maintained long enough or because the measurement is performed too soon (the particle continues deforming in the diverging region). On the other hand, the particle deformability may be limited, i.e., it may not be capable of further deformation even if we maintained a larger strain rate longer.

**Figure 12:**
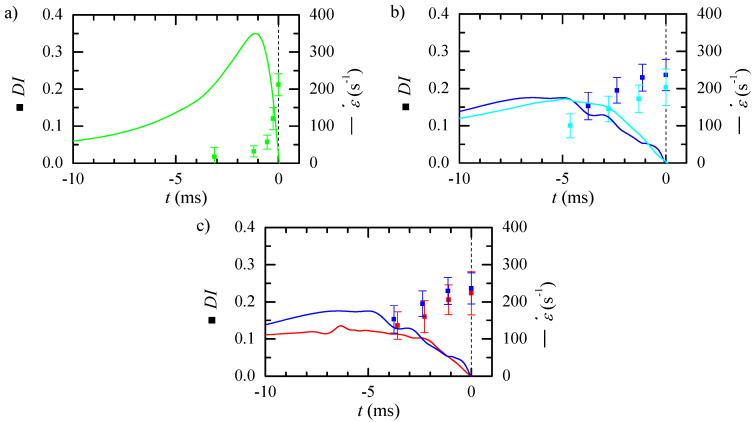
Temporal evolution of the particle deformation (symbols) and numerically calculated strain rate (lines) for nozzle 8 (**a**), 6 (blue) and 7 (cyan) (**b**), and 6 (blue) and 4 (red) (**c**). The flow rate was Q=5μL/min.

Particles deform as they travel with the flow, and the deformation at a particular section results from the strain rate upstream and how it is reached. We measured the deformation index at different axial positions within the observation window and used numerical simulations to estimate the time to reach the neck corresponding to that position. Figure 12 shows the particle deformation (symbols) and the numerical strain rate (lines) calculated in the previous section. A significant delay in the particle response is observed, which seems larger when the strain rate peak is more abrupt. For nozzle 8 (Figure 12a), the strain rate starts rising (exceeds 25 s${}^{-1}$) 20 ms before the neck. However, the particle reaction seems to initiate 3 ms before the neck. Though it deforms very fast, it does not seem to have finished deforming when it crosses the neck. Nozzles 6 (blue) and 7 (cyan) (Figure 12b) produce similar stress peaks, being that of the latter slightly delayed (approximately 1.6 ms). A similar delay is observed in the deformation trend for both nozzles, while for nozzle 6 the particle deformation seems to have reached its maximum at the neck (approximately 6.4 ms after the stress peak), for nozzle 7, it seems to be still increasing at that section. Nozzle 4 (red) (Figure 12c) has a lower stress peak but at the same position as nozzle 6 (blue). The resulting deformation is slightly smaller and appears to have reached a maximum at the neck.

**Figure 13:**
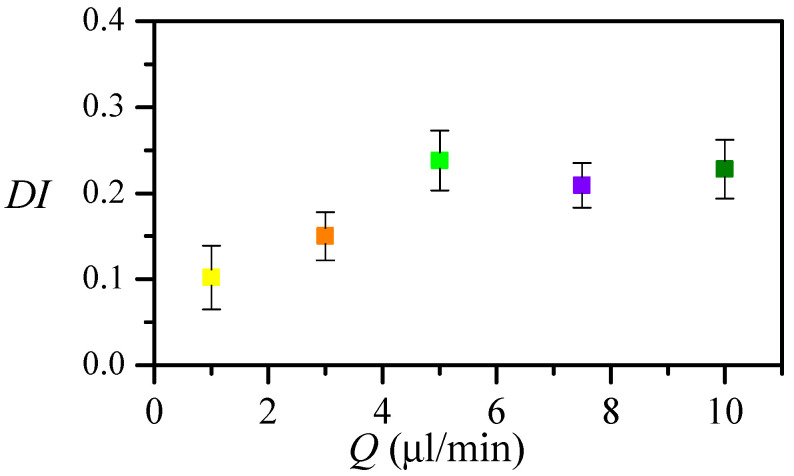
Deformation index DI at the neck versus the flow rate for nozzle number 8.

**Figure 14:**
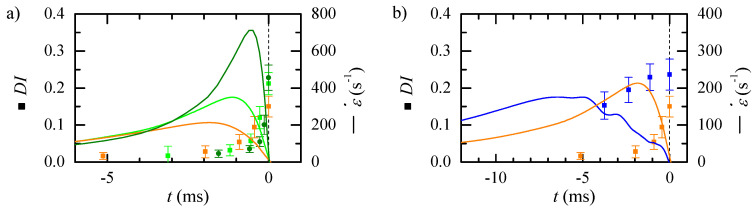
Temporal evolution of the particle deformation (symbols) and numerically calculated strain rate (lines) for: (**a**) nozzle 8, and different Q=3μL/min (orange), Q=5μL/min (light green) and Q=10μL/min (dark green); and (**b**) nozzle 6 and Q=5μL/min (blue) and nozzle 8 and Q=3μL/min (orange).

The flow rate allows controlling the strain rate peak, however, it also moves the peak position. We measured the particles DI at the neck for nozzle 8 at different flow rates (Figure 13). The particle DI increases with the flow rate up to Q=5μL/min, and then it remains almost constant. As there is a delay in the particle response, and, for this nozzle, the strain rate peak is very close to the neck, the deformation measured at that section is not the maximum. Figure 14a shows the temporal evolution of the deformation for three different flow rates and the strain rate evolution in the nozzle. Approximately the same low deformation is observed up to two milliseconds before reaching the neck. Then, the deformation starts raising earlier for the lower flow rate, for which the peak happens earlier. For the greater flow rate, the deformation occurs later and is sharper. Nevertheless, for all the flow rates, the response to the maximum stress seems to happen in the divergent region. Figure 14b compares the deformation for similar strain rates obtained using two nozzles and flow rates: nozzle 6 and Q=5μL/min (cyan) and nozzle 8 with flow rate Q=3μL/min (orange). The strain rate peak has to be applied more than 5 ms before the neck, to measure the maximum deformation at that section.

Our DI results for PDMS microparticles agreed with those reported in the literature for similar flow conditions and 2D rectangular microchannels (see, e.g., [5,12,37] and references therein).

## 4. Conclusions

Microcapsules are commonly used in several industrial applications. In many processes, their deformability is crucial for performing their function. For that reason, numerous methods have been developed to quantify this capability. Among them, the use of microfluidic devices is currently very popular due to the advances in their manufacturing techniques. Glass nozzles produced by fire-shaping are a low-cost alternative for evaluating microcapsules’ deformability flowing through constrictions. Mainly, these nozzles produce an extensional flow with a strain rate peak in the convergent region. Our numerical results show that the position and intensity of this peak can be estimated from a few geometrical parameters. Regardless of the nozzle shape, the maximum position occurs approximately at the section of 1.5 times the neck diameter. Its value and the time lag to the neck depend on the flow rate. The maximum ranges from 8 to 10 times the mean strain rate in the convergent region, and it occurs approximately 0.6 times the characteristic time $t_{c}$.

When studying the deformation of viscoelastic particles, such as PDMS, the response to the stress is not instantaneous. The duration and position of the peak should be considered as it affects the deformation measured at different sections. Nozzles fabricated on larger (or thicker) capillaries show longer necks, and so, the stress peak widens and moves away from the neck. Analyzing the microcapsule shape along the nozzle convergent region may allow the observation of both its deformation and recovery. On the other hand, nozzles fabricated from small and thin capillaries produce a very sharp peak close to the neck, and the microcapsule maximum deformation may occur beyond the neck. To measure the maximum deformation at the neck, the nozzle must produce the strain rate peak approximately 5 ms before it reaches that section.

## Figures and Tables

**Table 1:** Nozzles geometry and fabrication details.

	Nozzle Geometry			Capillary Geometry			Fabrication Parameters		
Nozzle	D [μm]	$L_{c}$ [mm]	$L_{n}$ [μm]	OD [mm]	ID [mm]	Wall	$r_{h}$ [mm]	$z_{h}$ [mm]	$t_{h}$ [s]
1	72 ± 2	2.61	672	3.7 ± 0.1	2.8 ± 0.1	Thick	3.5	15	900
2	65 ± 5	2.60	625	3.3 ± 0.1	2.8 ± 0.1	Thin	3.5	15	240
3	73 ± 3	1.61	475	3.3 ± 0.1	2.8 ± 0.1	Thin	4.5	15	600
4	63 ± 3	1.91	327	2.0 ± 0.1	1.0 ± 0.1	Thick	5.5	15	70
5	65 ± 1	1.62	314	2.0 ± 0.1	1.0 ± 0.1	Thick	6	15	120
6	62 ± 1	1.49	271	2.0 ± 0.1	1.6 ± 0.1	Thin	5.5	15	70
7	69 ± 7	1.12	220	2.0 ± 0.1	1.6 ± 0.1	Thin	6.6	15	480
8	72 ± 1	0.57	65	0.4 ± 0.04	0.3 ± 0.03	Thin	7.9	15	9

## Data Availability

Not applicable.
